# The Change in DMFT of Six-Grade Primary School Children in Shiraz two Years after Implementation of the National Oral Health Reform Plan

**DOI:** 10.30476/DENTJODS.2020.82850.1033

**Published:** 2020-09

**Authors:** Negin Zandi-Ghashghai, Aira Sabokseir, Ali Golkari

**Affiliations:** 1 Undergraduate Student, Student Research Committee, School of Dentistry, Shiraz University of Medical Sciences, Shiraz, Iran; 2 Dept. of Dental Public Health, School of Dentistry, Shiraz University of Medical Sciences, Shiraz, Iran

**Keywords:** Dental caries, Fissure sealants, Health plan, Implementation Iran, Topical fluorides

## Abstract

**Statement of the Problem:**

The oral health reform plan has been added to the Iran's health reform plan since the beginning of 2015. Evaluation of Iran’s oral health reform plan has rarely been conducted.

**Purpose:**

The aim of this study was to evaluate the change in DMFT among the six-grade primary school children of the city of Shiraz, two years after implementation of oral health reform plan.

**Materials and Method:**

A repeated cross-sectional study was conducted on six-grade primary school children of Shiraz in 2015 and 2017. About four hundred children were selected each year by cluster randomization sampling. The schools were randomly selected from three socioeconomically different types of schools including private schools, state schools in affluent areas, and state schools in deprived areas. The DMFT Index of selected children was compared between 2015 and 2017, and among three socioeconomically different areas. One-way ANOVA and Poisson regression tests were used for statistical analysis.

**Results:**

The mean DMFT of children was 1.47±1.83 in 2015 and 1.29±1.79 in 2017. There was significant difference in mean DMFT value between years 2015 and 2017 (*p*= 0.048). The percentage of children with untreated dental caries was 46% and 36.7% in 2015 and 2017 respectively. There was no statistically difference in DMFT of the three socioeconomically different schools.

**Conclusion:**

There has been significant improvement in DMFT of sixth grade school children of Shiraz two years after implementation of oral health reform plan.

## Introduction

Iran's Health Reform Plan (IHRP) is a national program approved and started in May 2014 to improve accessibility and quality of state health services comprehensively [ 1 ]. It started with focus on general health promotion, prevention, and hygiene. Given the close relationship between oral health and general health and the impact of oral health on general health [ 2 - 5 ], the Oral Health Reform Plan (OHRP) was added to the IHRP at the beginning of 2015 [ 6 ]. OHRP intend to improve oral health by providing education, prevention, and treatment services to target groups. Children under 14 years of age, pregnant women, and lactating mothers were the main groups to receive packages of dental services. Thousands of dentists, dental hygienists, and other trained auxiliaries were recruited to provide these services in two levels. Fluoride therapy, fissure sealant for first permanent molars, and restorative treatments for primary school children were part of these services and were provided free [ 7 , 8 ]. The first level was provided in "Health Houses" in rural areas and "Health Posts" in urban areas, and the next level services were provided in "Health Centers", all being part of Primary Health Care (PHC) Network [ 9 ].

Tooth decay is one of the most common oral diseases and has serious consequences for individuals and society, such as pain, dysfunction of the oral system and a decrease in the quality of life, the cost of treatment for the community and the loss of productivity of the individuals [ 10 ]. Studies conducted in different regions of Iran indicate a high prevalence of dental caries among young children and its significant differences among different regions of the country and between rural and urban areas [ 9 - 11 ]. The DMFT (decayed teeth, missing teeth due to caries, and filled teeth due to caries) Index of 12-year-old children was reported as 2.09 in 2012. The DMFT Index for age groups of 5-6, 15, 35-44, and 65-74 year olds were 5.16, 3.29, 13.20 and 25.71, respectively [ 9 ]. 

Scientific evidence shows that performing fluoride therapy and fissure sealant therapy are effective ways to prevent and stop dental caries [ 12 , 14 ]. These two services were highly appreciated by the OHRP authorities. The main outcome of the OHRP was reported to be the reduction of dental caries among primary school children due to the application of fissure sealant and topical fluoride. Department of Oral Health of the Iran's Ministry of Health has reported that the DMFT Index of 12-year-old children in Iran declined from 2.09 in 2012 to 1.84 in 2016, after implementation of OHRP [ 15 ].

Regular assessment of the OHRP is essential for assessing its efficiency and effectiveness, and helps identify the strengths and weaknesses of this program. In addition, the evaluation of this program can provide valuable evidence for health-care providers to take measures such as the continuation of the program or making corrective changes in the program. In this regard, a one-year evaluation of the fissure sealant program was conducted by the dental public health department of Shiraz Dental School in 2016 [ 16 ]. The success rate of fissure sealant in this one-year evaluation was low (only 47%) and many factors affected the quality of fissure sealants including the type of clinicians who applied the fissure sealant and the type of fissure sealant materials. The authors suggested the necessity of quality and quantity assessment of the program to achieve better results regarding the reduction of dental caries [ 16 ]. 

On the other hand, the increasing costs of health systems around the world have become one of the main concerns of managers and decision makers of health systems. The health system of Iran, like other countries, is faced with the challenge of rising costs. The implementation of the IHRP has led to an increase of about two times the tariffs for health care services, which put a large financial burden on health insurance organizations [ 17 ]. High cost is a major challenge to health promotion [ 18 ]. In OHRP, a large budget has been spent for infrastructure reconstruction of state dental centers, provision of equipment and dental materials, and the supply of human resources.

So far, little evidence exists regarding the efficacy and the effectiveness of the OHRP on the reduction of dental caries. The current study was designed to assess the possible changes in DMFT among six-grade primary school children of Shiraz two years after implementation of OHRP. Shiraz is the largest and most populated city in the south of Iran and has been one of the main centers of OHRP. 

## Materials and Method

A repeated cross-sectional study was conducted on sixth grade school children of the city of Shiraz in 2015 and 2017. Children were selected by cluster randomization sampling. The schools were randomly selected from three socioeconomically different types of schools including private schools, state schools in affluent areas, and state schools in deprived areas. Schools for children with special needs were not included. Ethical permission was obtained from the Ethical Committee of Shiraz University of Medical Sciences (SUMS) (# 1396-01-03-16434) and the educational head office of Fars province (# 97/2450793). 

In each selected school, all children aged between 12 years and zero months to 12 years and 11 months were included. The process of choosing schools and children was continued until 400 children were selected in 2015 and the same was repeated in 2017. Written consent forms were sent to their parents ahead of the examination day. Children whose parents did not provide written consent, or did not cooperate during the examinations, and those identified with systemic syndromes or diseases that could affect the DMFT Index were excluded from the study. 

Examinations were conducted in schools, in rooms other than the classroom (usually Health or Nursing Room) by a group of six calibrated final year dental students under supervision of a university lecturer. Students were called to the examination room one by one and were given the necessary explanations. The students were examined on a regular seat while their head was leaning against the back of the chair or wall, using headlight, disposable mirror, tongue blade, and disposable gloves. World Health Organization's recommended chart and criteria to record DMFT for screening studies were used [ 19 ]. 

After descriptive assessment of the DMFT Index and its components (D: decayed tooth, M: missing due to caries, F: filling due to caries), one-way ANOVA test was used to assess the possible differences among the three socioeconomically groups. This was done in each assessment year separately. In addition, Poisson regression tests were used to assess the changes in DMFT, DT, MT, and FT from 2015 to 2017. The IBM SPSS Software (version 22) was used for data analysis. The significance level was set at α = 0.05.

## Results

A total of 363 six-grade schoolchildren were included in the final analysis in 2015 (response rate= 91%), and 398 were included in 2017 (response rate= 99%). The descriptive statistics of DMFT and its components are given in Table 1.

**Table 1:** The descriptive statistics of DMFT and its components in the years 2015 and 2017

Year	School type	DT		MT		FT		DMFT	
2015	Private	0.99 (±1.30)	1.14 (±1.61) p*=0.052	0.00 (±0.00)	0.02 (± 0.25) p*=0.030†	0.37 (±0.96)	0.31 (±0.88) p*=0.016†	1.35 (±1.55)	1.47 (±1.83) p*=0.647
	State in affluent areas	0.87 (±1.55)		0.10 (±0.56)		0.54 (±1.00)		1.49 (±2.04)	
	State in deprived areas	1.35 (±1.82)		0.01 (±0.11)		0.19 (±0.73)		1.55 (±1.96)	
2017	Private	0.54 (±1.20)	0.81 (±1.51)p*=0.141	0.00 (±0.00)	0.03 (± 0.22) p*=0.129	0.54 (±1.17)	0.46 (±1.01) p*=0.013†	1.09 (±1.68)	1.29 (±1.79) p*=0.278
	State in affluent areas	0.86 (±1.50)		0.02 (±0.17)		0.54 (±1.07)		1.42 (±1.79)	
	State in deprived areas	0.95 (±1.75)		0.06 (±0.38)		0.19 (±0.62)		1.20 (±1.91)	
Significance level Comparing 2015 and 2017		p**<0.001†		p**=0.782		p**=0.002†		p**=0.048†	

* *p*: Significance level of the difference among three socioeconomically different schools

** *p*: Significance level of the difference between 2015 and 2017 measurements

† : Statistically significant at α= 0.05

In 2015, the overall percentage of children with DMFT > 0 was 52.6 with the mean DMFT of 1.47 ± 1.83. It was found that 77.3% of DMFT was related to decayed teeth, while only 21.3% of it was related to filled teeth. The overall percentage of children with untreated dental caries in their permanent teeth was 46%. The percentage of children with DMFT> 0 in private schools, state schools in affluent areas, state schools in deprived areas were 55.6%, 44.4% and 53.3% with the mean DMFT values of 1.35±1.55, 1.50± 2.03, 1.55±1.96 respectively (*p*= 0.647). 

In 2017, the overall percentage of children with DMFT> 0 was 49.5 with the mean DMFT of 1.29±1.79. It was found that 62.7% of DMFT was related to decayed teeth, while 35.4% of it was related to filled teeth. The overall percentage of children with untreated dental caries in their permanent teeth was 36.7%. The percentage of children with DMFT > 0 in private schools, state schools in affluent areas, state schools in deprived areas were 43.5%, 54.0%, and 45.3% with the mean DMFT values of 1.09±1.68, 1.42±1.79, 1.20±1.91 respectively (*p*= 0.278). 

The decrease in DMFT value from 1.47 in 2015 to 1.29 in 2017 was statistically significant (*p*= 0.048), while there was no statistically difference in DMFT of the three socioeconomically different schools (Table 1). 

The share of D of DMFT among six-grade schoolchildren reduced from 77.3% to 62.7% from 2015 to 2017. In the meantime, the share of F of DMFT increased from 21.3% to 35.4%. The share of M also had a change from 1.4% in 2015 to 1.9% in 2017. Furthermore, the overall percentage of children with untreated dental caries in their permanent teeth decreased from 46% to 36.7%.

## Discussion

The aim of this study was to conduct an evaluation of OHRP by assessing the probable changes in DMFT and its components among six-grade primary school children of Shiraz, two years after implementation of the OHRP. Although two years might be considered a short period for assessment of such a plan, an early evaluation could help the authorities recognize how they should carry on the OHRP. 

The results of the current study showed that the mean DMFT of the six-grade children in Shiraz decreased from 1.47 in 2015 to 1.29 in 2017. Although the 0.18 decrease in DMFT might sound a small number, it should be consider that it compose 12% of the DMFT at the beginning of the study period, which was relatively low itself. Therefore, it was not surprising that this amount of reduction was statistically significant. There was no significant difference regarding the mean DMFT between socioeconomic groups in either 2015 or 2017. However, there were some differences in components of DMFT in each given year. 

The mean DMFT value of each of the three socioeconomically different schoolchildren groups decreased from 2015 to 2017 and this decline was more prominent in schoolchildren of deprived areas. There was significant difference between groups regarding D, M, and F in 2015, whereas, in 2017, the difference of D and M between groups was not significant. 

The Department of Oral Health of the Iran's Ministry of Health has estimated the reduction of DMFT (2.09 to 1.84) from 2012 to 2016 for the whole country [ 15 ]. The DMFT Index in the current study has also showed a decrease from 1.47 to 1.29. It appears that so far, OHRP has been successful in reduction of dental caries among the 12-year-old children as one of its main target groups. OHRP has also been able to reduce the inequalities in the treatment/care rate (decrease in D and increase in F) of socioeconomically different children. 

This was the first study in its kind to assess the outcomes of OHRP. There has been no other study regarding the assessment of OHRP in other cities of Iran. Therefore, a comparison of the results of this study with other cities of Iran was impossible. 

The DMFT Index has decreased after two years of implementing the OHRP. However, it might be said that dental caries are affected by many factors, therefore, the credit might not be all for the OHRP. However, another study that also conducted in the city of Shiraz, using examination and reporting methods similar to the current study, reported that the DMFT of the 11-year-old schoolchildren was 1.4 in 2009 [ 20 ]. That finding is close to the DMFT reported for 12-year-olds in 2015 in the current study. This proximity shows that the DMFT of this age group was almost steady for years before the implementation of the OHRP. In other words, as the DMFT seems not changed from 2009 to 2015, and it has significantly changed from 2015 to 2017, we could assume that the change could be related to OHRP. 

The current study was conducted only in one city and in a short period (two years), using only one index (DMFT); however, considering the size of resources that OHRP has used, it is expected to observe more positive effects on Iranian's oral health. Regular and periodic assessments of OHRP, preferable multi-center studies, are recommended to evaluate the efficiency and effectiveness of the program. 

## Conclusion

The mean DMFT of six-grade primary school children of Shiraz has significantly decreased by 12%, two years after implementation of OHRP. This decline was more prominent in schoolchildren of deprived areas (23%). Iran's oral health policy makers should promote regular multi-center assessment of OHRP outcomes in order to employ appropriate strategy. 
